# Acceptability and Feasibility of HPV Self-Sampling as an Alternative Primary Cervical Cancer Screening in Under-Screened Population Groups: A Cross-Sectional Study

**DOI:** 10.3390/ijerph17176245

**Published:** 2020-08-27

**Authors:** Eliza Lai-Yi Wong, Annie Wai-Ling Cheung, Amy Yuen-Kwan Wong, Paul Kay-Sheung Chan

**Affiliations:** 1 Centre for Health Systems and Policy Research, JC School of Public Health and Primary Care, Faculty of Medicine, The Chinese University of Hong Kong, Hong Kong, China; anniewlcheung@cuhk.edu.hk (A.W.-L.C.); amy.wong@cuhk.edu.hk (A.Y.-K.W.); 2 Department of Microbiology, Faculty of Medicine, The Chinese University of Hong Kong, Hong Kong, China; paulkschan@cuhk.edu.hk

**Keywords:** cervical cancer prevention, HPV self-sampling, under-screened population, cervical screening preference, women’s health promotion

## Abstract

*Background:* Cervical cancer is one of the most common cancers in women and about 90% of cervical cancer can be reduced by regular screening. The Pap smear has been well in place as a primary cervical screening method since 1950s; however, coverage is still not optimal. This study explored the feasibility of HPV self-sampling in two under-screened population groups in Hong Kong (HK): never screened and not regularly screened females, to estimate the uptake rate and preference rate in the future. *Materials and Methods:* This was a cross-sectional study to explore the acceptability and feasibility of HPV self-sampling in two age groups: aged 25–35 and aged ≥45, which were reported as the highest proportion of the under-screened population in HK between 2017 and 2018. The study invited eligible women from an HPV study cohort to perform HPV self-sampling at home by themselves. The number of specimens returned from participants was recorded and used to determine the feasibility of HPV self-sampling in the community. The participants were asked to fill in the questionnaires before and after HPV self-sampling to indicate their attitudes, acceptability, and future preference for HPV self-sampling as an acceptable alternative primary cervical cancer screening method. *Results:* A total of 177 subjects participated in the present study and have achieved a good overall uptake rate of 73% (129/177) who returned the self-collected cervicovaginal sample for HPV testing. Among the under-screened population, there was a higher response rate in aged ≥45 than those aged 25–35. The findings also revealed that women who were under-screened, including those who have never been screened, were more likely to prefer HPV self-sampling than those who had regular screening. This study found that the acceptability of HPV self-sampling was fairly positive among the respondents. The findings also indicated that HPV self-sampling was not only beneficial to enhance their health awareness but also to promote the cervical cancer screening uptake rate, especially among the under-screened or never screened populations. *Conclusions:* HPV self-sampling would be a solution to overcome the perceived barriers in clinician-based screening. The findings also indicated that it could be feasible to use as an alternative primary cervical cancer screening.

## 1. Introduction

According to the World Health Organization, cervical cancer is one of the most common cancers in women and about 90% of cervical cancer can be reduced by regular screening. The association between Human Papillomavirus (HPV) and cervical cancer is well established. Due to the slow growing nature of HPV, cervical cancer is regarded as highly preventable and treatable if it can be detected earlier [1]. The Pap smear has been well in place as a primary cervical screening method since 1950s; however, coverage is still not optimal. The uptake rates of cervical screening vary in different jurisdictions: 21.4% of women aged ≥21 in China in 2014 [2], 50.7% of women aged 30 to 59 in the European Union in 2013 [3], 74.0% of women aged 25 to 69 in Canada in 2017 [4], and 81.0% of women aged 21 to 65 in the United States in 2015 [5]. In recent decades, Hong Kong (HK) has also put effort into promoting cervical screening but still has a considerably low coverage, where the uptake rate was less than half (47.6%) in 2015 [6]. Women aged 25–34 (57%) had the highest proportion of never had cervical screening, while those aged 25–34 (52%) and aged 55–64 (57%) had a comparatively higher proportion of not having regular cervical screening [6]. The most common reasons of overdue or delaying the Pap smear including fear and lack of control, fear of pain, embarrassment, have no time, not relevant to me, and stranger examination in a non-intimate setting [7,8,9].

With the emerging of supportive findings in the causal relationship between persistent infection of high risk HPV and the development of cervical cancer, additive testing for HPV was implemented as co- or standalone- testing so as to increase the sensitivity and specificity of population-based cervical screening [10,11]. To overcome the barrier of the Pap smear, an increasing number of studies have been exploring whether adopting a HPV self-sampling method would lower the threshold for women to participate in cervical cancer screening since the end of 1990s [12,13]. Recently, a meta-analysis was conducted to provide an update overview of the reliability and effectiveness of HPV self-sampling on the participation rates in under-screened populations [14]. Some studies have reported a positive impact of different invitation strategies on the uptake rate of HPV self-sampling. HPV testing has been implemented as a primary test in which HPV self-sampling is offered as an option for population-based organised screening in four countries including The Netherlands in 2017 [15] for those who find it difficult to have a smear test; Australia in 2017 [16] for those who are 30 years or over and have never had the Pap smear or are at least two years overdue for screening; Malaysia in 2019 [17] for women aged 30–49 years to collect samples using a vaginal self-swab; and Finland in 2019 [18] offering self-sampling to non-attendees of the Finnish cervical screening programme.

In HK, the Guidelines for Cervical Cancer Prevention of The Hong Kong College of Obstetricians and Gynaecologists were revised in 2016 [19] by adding a management option for the HPV standalone test; however, to date, local physicians are still keen on keeping the traditional practice by suggesting the Pap smear as the primary screening method to women. A local study suggested that women would reduce hesitation to undergo cervical screening if the HPV self-sampling screening were introduced [9]. It also predicted that some of the women would shift to the self-collected HPV sampling instead of the clinician-collected Pap smear testing performed in clinics. If so, the screening rate would be increased eventually, hence, more treatable cases could be detached early. Thus, the government has a role to help monitor women’s health and to embed an alternative option in place. However, it remains unclear the response rate of HPV self-sampling per se in different socioeconomic groups of the under-screened population and the number of those who attend the Pap smear that would shift to HPV self-sampling. In this study, we explored the feasibility of HPV self-sampling in two under-screened population groups to estimate the uptake rate and preference rate in the future. We hypothesised that HPV self-sampling could improve the uptake rate of cervical cancer screening and yield high preference to support HPV self-sampling as an alternative primary screening in future.

## 2. Methods

### 2.1. Study Designs

This was a cross-sectional study to explore the acceptability and feasibility of HPV self-sampling in two age groups: aged 25–35 and aged ≥45, which were reported as the highest proportion of under-screened population in HK between 2017 and 2018. It was an ongoing community-based project to promote using HPV self-sampling as a cervical screening tool in the community and invite women to do HPV self-sampling by themselves at home. If the participants agreed, their uptake rate of HPV self-sampling and cervical cancer screening records would be tracked in subsequent years. This paper aimed to report the return rate and feedback of the participants after the first attempt of conducting HPV self-sampling in the study population.

### 2.2. Study Population

Participants were recruited from two different sources for corresponding types of the under-screened population. First source: women aged 25–35 were recruited from graduates of one of the selected local universities through the HPV Vaccination Campaign Registry or their peer network. A total of 1328 HPV-vaccinated female registered with the campaign were approached by a telephone survey using a structural questionnaire with a response rate of 49% (651/1328), in which 117 participants indicated their interest to perform the HPV self-sampling. Upon agreeing to participate in the study, participants were given guidance on the use of HPV self-sampling kits by phone first and an HPV self-sampling package was sent to their mailing address for self-completion. A complete written informed consent and a post-questionnaire survey were returned, together with the self-collected samples once they had performed the HPV self-sampling. Second source: women aged ≥45 were recruited through community centres in Hong Kong. Most subjects recruited from this group would have not received the HPV vaccination as the vaccine was not available until 2014 in Hong Kong. With the facilitation of local district officers, interested participants from the community were invited to join the study with a face-to-face interview and a take home HPV self-sampling package was provided at the end. Both study groups were also presented with a link to video instructions to perform the self-sampling at home.

Due to the applicability to perform the HPV self-sampling, women who were pregnant, or possibly pregnant; with hysterectomy; with cancers of the reproductive system; with exposure to hormonal therapy (long-term steroids user); and with immune function disease or on immune therapy were excluded from the studies. Immunocompromised is considered to be the prerequisite for disease progression in such a way that it essentially facilitates persistent hr-HPV infection and cervical lesion progression. In order to make a generalization of the populations, those who are almost certain to acquire HPV infection were excluded from the study.

### 2.3. HPV Self-Sampling

For HPV self-sampling, the consented participants were provided with the self-sampling package either on the recruitment site or by mailing upon the request of participants. The package included a HPV self-sampling kit, written instructions for use in Chinese with diagrams, and a verbal explanation so that the materials and procedure of self-sampling were fully understood. The Evalyn^®^ Brush (Rovers Medical Devices B.V., Oss, The Netherlands) was the self-sampling brush device used in the study to collect a cervicovaginal sample for subsequent high-risk HPV testing. The instructions described how to insert the brush device into the vagina to a depth of at least 2 inches and make 3–5 full rotations, then, remove the device from the vagina and place the cap back on before putting inside the packaging. The instructions also suggested the method of returning the specimens using the pre-stamped self-addressed envelope to our office. For those participants who did not want to mail back their specimens, there was an option for them to return them to our research team in person at the community clinic.

### 2.4. Laboratory Test for HPV Detection and Genotyping

The self-collected cervicovaginal samples were transported to the laboratory for HPV testing with DNA extraction using the QIAamp DNA Mini Kit [20]. Two recently developed novel PCR-based Next-Generation Sequencing (NGS) assays targeting the consensus regions of the HPV L1 open reading frame were used to detect and identify the full spectrum of Human Papillomaviruses including Alpha-, Beta- and Gamma-HPV types, as described previously [21]. Briefly, a pair of unique 12-bp barcodes were introduced to the PCR amplicon by forward and reverse primers. Successful amplicons with predicted fragment sizes were pooled at approximately equal molar DNA concentrations and sequenced on an Illumina MiSeq [22] using 150-bp paired-end reads. The demultiplexed paired-end Illumina short reads passing the quality filter (≥Q20 and ≥50-bp) were merged into single reads using FLASh v1.2.11 and blasted against the genomes online database (gold) papilloma virus (PV) reference database using UPARSE software. Our PV reference database contains 387 fully characterized human (n = 225) and animal (n = 162) PV types, and 467 potential novel partial PV sequences. An operation taxonomic unit (OTU) count table giving the number of reads per sample per OTU was created using a 95% identity threshold using in-house developed scripts. The OTU taxonomy was classified at the type level based on sequence homology to the reference database; if OTUs hit the reference database with ≥90% identities to a characterized PV type, it indicated known viruses were presented, while those with 60–89% identities were regarded as ‘uncharacterized’ types and assigned with a unique identity. An HPV type was considered positive if the reads were ≥50.12 Alpha-HPV types (HPV16, 18, 31, 33, 35, 39, 45, 51, 52, 56, 58, 59) classified as “carcinogenic to humans” (Group 1) and were considered as high-risk HPV types in this study. 

### 2.5. Outcome Measure

The number of specimens returned from participants were recorded and used to determine the feasibility of HPV self-sampling in the community. The participants were asked to fill in the questionnaires before and after HPV self-sampling to indicate their attitudes, acceptability, and preference for HPV self-sampling as an acceptable alternative primary cervical cancer screening method in the future.

All participants completed the baseline standardised questionnaire, which included information on age, sex, lifestyle risk factors (e.g., early sexual debut, having more lifetime sexual partners, postcoital bleeding, urethritis, Sexually Transmitted Disease (STD), frequent sexual activity, and no use of contraceptive method), and cervical cancer screening behaviour. Laboratory results of genotypes of HPV self-sampling including high risk (hr) and low risk (lr) were also reported.

### 2.6. Statistical Analysis

Descriptive statistics (numbers and proportions) were used to report the socioeconomic characteristics of the women who received the HPV self-sampling in the studies. The response rate (uptake rate) of HPV self-sampling (specimens successfully returned for laboratory testing) in each age group and the overall and subgroup proportions according to their cervical cancer screening behaviour were reported. The prevalence of HPV infection with different genotypes (HR or LR) of HPV was reported. Furthermore, to determine if the uptake rate of HPV self-sampling was modified by the socioeconomic status and lifestyle risk factors, both univariate and multiple regression models were applied. Chi-square test or Fisher’s exact test and the t-test or ANOVA were used when comparing categorical and continuous variables, respectively. Univariate logistic regressions were further applied to study the adjusted association of socioeconomic status and lifestyle risk factors (e.g., cervical cancer screening patterns and sexual behaviour, etc.) if necessary. All analyses were performed using IBM SPSS Statistics Version 24 (IBM, Armonk, NY, USA) [23] and *p*-value < 0.05 was considered to be statistically significant.

### 2.7. Ethical Consideration

The study was conducted in accordance with the Declaration of Helsinki and ethical approvals were obtained from The Joint Chinese University of Hong Kong–New Territories East Cluster Clinical Research Ethics Committee (The Joint CUHK-NTEC CREC). For the respondents who agreed to participate in the study using the self-collected HPV testing, written consent was obtained. All the respondents were informed of the study purpose, study details, research procedures, and its potential risks and benefit, and the right of refusal or withdrawal from the study. All information collected from respondents were treated with strict confidentiality.

## 3. Results

### 3.1. Demographic Characteristics

A total of 177 participants were recruited between September 2016 and September 2017 for those aged ≥45 (60 cases) and between March 2018 and November 2018 for those aged 25–35 (117 cases). The demographic characteristics of the two groups are shown in Table 1. In addition, nearly half of the women (48%, 29/60) aged ≥45 had either never done the cervical cancer screening (Pap smear) or were under-screened (those who had not had the Pap smear in the past 5 years). For women aged 25–35, the proportion of never screened and under-screened (those who had not had the Pap smear in the past 3 years) was 57% (67/117), which is relatively higher than those aged ≥45.

### 3.2. Response Rate of HPV Self-Sampling

**Figure 1:**
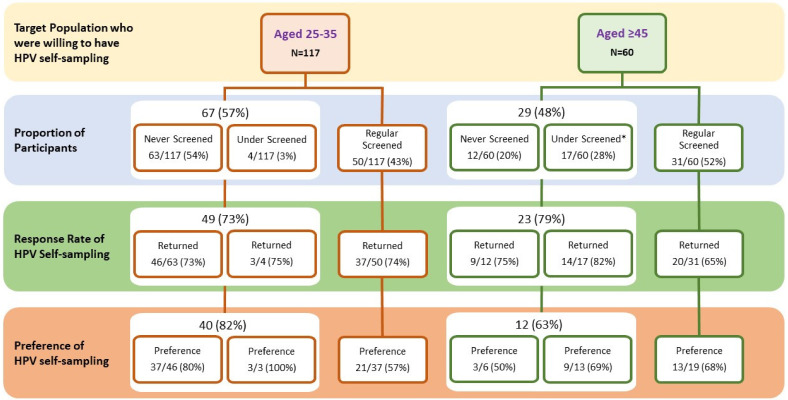
Response rate of HPV self-sampling and preference for HPV self-sampling by cervical cancer screening behaviour of participants. “Under Screened” was defined as not having Pap smear screening in the past 3 years for those aged 25–35, while it was defined as not having Pap smear screening in the past 5 years for those aged ≥45. “Never screening” was defined as never having the Pap smear before. “Response rate of HPV self-sampling” means the proportions of returning HPV specimens after performing HPV self-sampling. Preference for HPV self-sampling means willingness to use HPV self-sampling as an alternative primary screening for cervical cancer in the future after trying HPV self-sampling. A few numbers were not added up to total due to missing values.

Among the 177 participants, those who showed initial interest in HPV self-sampling were invited to perform HPV testing at home by taking the self-sampling package away from the recruitment site/mailing to their address. At least three reminders were sent via telephone if the participant had not returned their HPV specimen. The most common reasons for not returning included having no time, not confident in the sampling procedure, and some might have simply failed in their attempt to collect the sample. The overall response rate of HPV self-sampling among the participants was 73% (129/177). The response rates among the two age groups were similar—74% for aged 25–35, while 72% for aged ≥45. The uptake rate was considerably higher among those who had never been screened or were under-screened (75%; 72/96) than those with regular cervical cancer screening (70%; 57/81). The details are shown in Figure 1.

### 3.3. Prevalence of HPV Infection

HPV infection was detected in 3 of the 129 respondents (2.3%). The overall prevalence of HPV infection among women aged ≥45 was 4.7% (2/43), which was higher than those aged 25–35 (1.2%, 1/86). Among the three HPV-positive cases, they were all either under-screened or never screened. Furthermore, the prevalence of hr-HPV genotype infection among respondents ≥45 was 100% but none was detected among women aged 25–35 (only the lr-HPV genotype was found). However, the low hr-HPV prevalence among the younger age group may be due to the protection of the HPV vaccination.

### 3.4. Factors Associated with the Uptake of HPV Self-Sampling

The results showed that only having religious belief was significantly associated with the HPV self-sampling uptake among those aged 25–35 years. Overall, women who were under-screened or never screened with the Pap smear (75%, 72/96) were generally more likely to return the HPV self-sampling kit than those had regular Pap screening (70.4%, 57/81). In particular, under-screened/never screened women aged ≥45 were more likely to uptake HPV self-sampling, whereas women aged 25–35 had a higher uptake rate among those who were regularly screened than under-screened. No statistical significance was detected in either under-screened/never screened women or those with regular screening. The details are shown in Table 2.

### 3.5. Acceptability Towards HPV Self-Sampling

**Figure 2:**
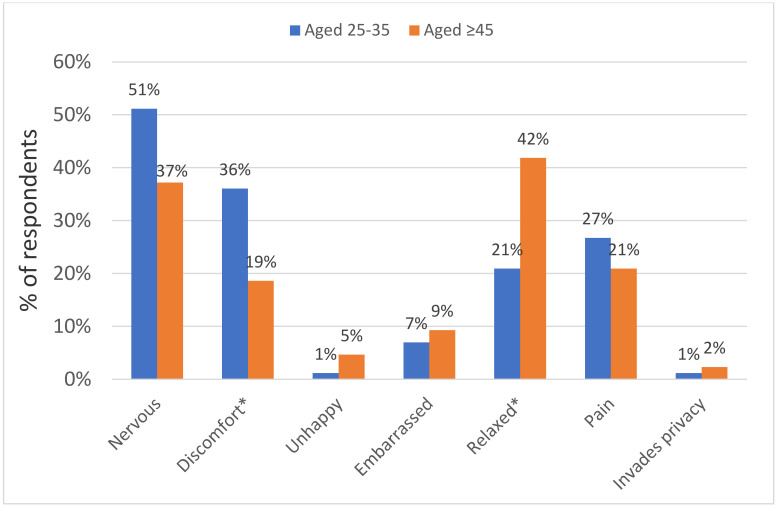
Feelings towards HPV self-sampling after the sampling procedure among the two age groups. % was calculated based on women who scored 4 or above on the Likert scale from 1—strongly disagree to 5—strongly agree. There was a total of 86 participants in the group aged 25–35 (1 missing value in invades privacy dimension) and a total of 43 participants in the group aged ≥ 45. Chi-square/Fisher exact tests were used to compare the differences among the two age groups; * *p*-value < 0.05.

The acceptability of HPV self-sampling was measured in five domains using the post-questionnaire: (1) ease of use; (2) trusting the test results; (3) handling procedure; (4) beneficial to health; and (5) convenience. Overall, more than half of the respondents gave a positive response (scored 4 or above on the Likert scale) in all five domains. The majority of respondents reported that it was convenient (86% and 95%) and beneficial to health (94% and 95%) among both aged 25–35 and aged ≥45, respectively. However, there was no statistical difference between the two groups. The details are shown in Table 3. Feelings towards HPV self-sampling after the sampling procedure were captured. The results showed that respondents experienced less discomfort and felt more relaxed during the sampling procedure among the aged ≥ 45 than aged 25–35 years (Figure 2). Furthermore, when asking for overall comments on HPV self-sampling with an open-ended option, most have given positive feedback that it was convenience, time-saving, and flexible but also on the down side, with lack of confidence in self-collection and concerns about the test accuracy, which are supported in our acceptability analysis (Table 3).

### 3.6. Future Preference for HPV Self-Sampling as an Alternative Primary Screening for Cervical Cancer

In the overall study population, the proportion of preference towards HPV self-sampling as an alternative primary screening for cervical cancer was 69% (86/124). For analysis, options with not able to decide between the Pap smear and HPV self-sampling or preferred both were also included in the preference for HPV self-sampling in the present study. Preference among age groups was presented in Figure 1. However, there was no statistically significant difference (*p* > 0.05) between the proportions of HPV self-sampling preference among the two study groups. The overall preference for HPV self-sampling with different screening patterns among those who had completed the follow-up study is shown in Figure 1. Regardless of their age, women who had never screened or were under-screened were more likely to prefer HPV self-sampling than those with regular screening.

## 4. Discussion

This is the first study to explore uptake rate of HPV self-sampling and its acceptability and feasibility among the under-screened population in HK. This study’s findings report an encouraging uptake rate of HPV self-sampling with 73%, considerably higher in the under-screened/never screened population, in particular, among aged ≥ 45. This study found that the acceptability of HPV self-sampling was very positive among both women aged 25–35 and aged ≥45, indicating that it could be a solution to overcome the barrier of the Pap smear and feasible to use as an alternative cervical cancer screening to increase the coverage of cervical cancer screening. 

### 4.1. HPV Self-Sampling

High response rate was observed in under-screened and never screened populations, indicating that the acceptability and feasibility of HPV self-sampling as an alternative primary screening are promising in Hong Kong. Our findings align with other jurisdictions that focused on the feasibility and evaluation on HPV self-sampling in cervical cancer screening due to its high sensitivity, high acceptability, and cost-effectiveness in health settings [24,25,26]. In addition to the experiences of other jurisdictions, our study provided substantial information for decision-making in health policy, implementation, and evaluation of the national cervical cancer prevention programme in Hong Kong.

### 4.2. Factors Associated with the Uptake of HPV Self-Sampling

In our study, religious belief was significantly associated with the high uptake rate of HPV self-sampling among younger women. In other words, religion significantly determined knowledge and prevention behaviour that influenced their decision in the uptake of cervical cancer screening. As not many studies have explored factors associated with the uptake of HPV self-sampling but Pap smear testing (e.g., [27]), it was difficult to make a direct comparison with these studies. Nevertheless, it is worth noting that age, education level, household income, smoking and drinking frequency, and job status were associated with the uptake of cervical cancer screening [26]. Hence, more research on the uptake of HPV self-sampling and its associated factors is needed.

### 4.3. Acceptability of HPV Self-Sampling

Perceptions and experience of HPV self-sampling were evaluated in five domains, including ease of use, handling procedure, trusting the test results, beneficial to health, and convenience, which all have been considered as key attributes of acceptability [28]. Overall, the majority of respondents (>80%) reported that it was convenient, easy to handle, and had confidence in handling the procedure; these results were in line with previous studies [29,30,31,32]. However, the HPV self-sampling kits were used differently across studies and several factors may have an impact on user’s acceptability and preference towards self-sampling such as sociodemographics, religious beliefs, and race/ethnicity or cultural differences [33]. Thus, the underlying variations on the acceptability of self-sampling across studies should be explored in order to incorporate self-sampling as a screening modality for the national cervical cancer prevention programme.

Among the under-screened women with preference for HPV self-sampling, a considerably larger proportion was found among those aged 25–35 than those aged ≥ 45, indicating that there was an age-related acceptability of HPV self-sampling in cervical cancer screening [34]. Our results showed that younger women were less confident in their own competency and had more doubts on trusting the test results. One reason could be that the level of education was associated with confidence, where women with higher education, considering our younger age group which all had an education of tertiary or above, were less confident in collecting the self-sampling [33]. Several explanations for that could be those with higher education were more doubtful in their abilities or simply because they were comfortable for the professional to carry out the test than themselves. However, a larger sample size is needed, hence, reducing the chance of sampling error occurring for future study.

### 4.4. Barriers to Cervical Cancer Screening: Pap Smear vs. HPV Self-Sampling

The most common reasons for women not attending the clinician-based cervical cancer screening included fear of pain, lack of control, embarrassment, privacy concerns, and unpleasant experience of screening in the past [9]. The present study has revealed that only less than 8% of women felt embarrassed, while nearly 30% felt that they were relaxed during self-sampling, indicating that these common barriers to clinician-based cervical cancer screening can be effectively overcome. Almost all women did not find that self-sampling invaded their privacy, which was supported in many studies where more privacy was found in performing self-sampling than clinician-based Pap smear testing [30,35]. However, lack of confidence in their own competency and not trusting the method were explored as barriers to self-sampling [36,37]. Our study showed that despite over 80% of respondents being confidence in handling the procedure, only around half indicated that they had collected the samples correctly. Therefore, raising public awareness and education on HPV to prevent cervical cancer, in particular the introduction of self-sampling, is required.

### 4.5. Current Situation: HPV Self-Sampling as an Alternative Primary Screening

According to the latest WHO guidelines for the prevention and control of cervical cancer [38], HPV testing is suggested as primary testing to screen women for cervical cancer prevention with intervals of no less than 5 years. This could be a more cost-effective strategy in strengthening healthcare systems. The Netherlands was the first country to implement high-risk HPV testing with the option of self-sampling in primary screening at the national level. Studies have also shown that the benefits of HPV testing in screening programmes are evidenced, which not only to increase the length of screening interval but its high sensitivity has the potential to increase detection [14]. Thus, self-sampling would be a solution to overcome the perceived barriers in clinician-based screening. Among the three HPV positive cases found in the study, they were all either under-screened or never screened, indicating that HPV self-sampling was not only beneficial to enhance their health awareness but also to promote the cervical cancer screening uptake rate.

Overall, around 70% of the respondents reported preference for HPV self-sampling in our study, which is consistent or even more feasible than other studies, where the pooled estimate of women who preferred self-sampling was 59% [28]. Furthermore, under-screened women were more likely to prefer self-sampling than those with regular screening [33,39]. Comparatively, our results revealed that more than two-thirds of respondents who were under-screened (including never screened) would prefer HPV self-sampling in the future. Screening with HPV self-sampling is thus considered to be a feasible and practical approach to increase screening coverage. It is proven that the incorporation of HPV self-sampling strategies into screening programmes not only can be used to increase screening coverage but also can be offered as an alternative screening tool to all women [40].

## 5. Strengths and Limitations of this Study

This is among the first to conduct HPV self-sampling in the under-screened population in Hong Kong and is an on-going project to keep track of the uptake rate of HPV self-sampling. Such data provide support for evidence-based decisions in health policy making to incorporate self-sampling strategies into the organised cervical screening programme.

Our study forms a basis for promoting cervical cancer screening and increasing the knowledge of HPV and cervical cancer. Lack of self-competency and testing anxiety arose with uncertainties while women tested in their homes, as HPV self-sampling was still fairly new to the majority of Hong Kong women, thus public education is required.

A larger study is needed to improve the validity of the research and to provide results generalisable to the Hong Kong population.

## 6. Conclusions

HPV self-sampling as an alternative primary cervical cancer screening is feasible and acceptable to under-screened women in Hong Kong. It could effectively increase screening coverage among those under-screened populations. Considering the local organised cervical cancer prevention programme in Hong Kong, the HKCOG Guidelines for Cervical Cancer Prevention and Screening [19] suggested that HPV testing could be used as primary screening, either as part of co-testing with cytology or as a standalone test in primary screening. However, while maintaining co-testing can improve the sensitivity for precancerous lesions, it also creates significant resources burden and cost implications. If HPV self-sampling can be used as an alternative primary screening, it could undoubtedly overcome these implications.

## Figures and Tables

**Table 1:** Demographic characteristics among those who responded to the study.

	Aged 25–35 N = 117 (%)	Aged ≥ 45 N = 60 (%)	Total N = 177 (%)
Age			
Mean {S.D.; Range}	30 {1.8; 27–35}	57 {5.8; 45–68}	39 {13.1; 27–68}
Marital status			
Single/divorced/widow	70 (59.8)	11 (18.3)	81 (45.8)
Married/cohabited	47 (40.2)	49 (81.7)	96 (54.2)
Highest Education Attainment			
Primary or below	---	8 (13.3)	8 (4.5)
Secondary	---	39 (65.0)	39 (22.0)
Tertiary or above	117 (100.0)	13 (21.7)	130 (73.5)
Religious Belief			
Yes	34 (29.1)	23 (38.3)	57 (32.2)
No	83 (70.9)	37 (61.7)	120 (67.8)
Employment status			
Full-time/part-time	116 (99.1)	22 (36.7)	138 (78.0)
Unemployed	0 (0.0)	4 (6.7)	4 (2.2)
Retired/Housewife	1 (0.9)	34 (56.7)	35 (19.8)
Screening patterns (Pap smear)			
Never screened	63 (54.0)	12 (20.0)	75 (42.4)
Under-screened	4 (3.0)	17 (28.0)	21 (11.9)
Regularly screened	50 (43.0)	31 (52.0)	81 (45.7)

**Table 2:** Factors associated with uptake of HPV self-sampling.

	Returned HPV Self-Sampling Kit for Laboratory Test					
	Aged 25–35 N = 86 (Row%)	*p*-Value *	Aged ≥ 45 N = 43 (Row%)	*p*-Value *	Total N = 129 (Row%)	*p*-Value *
**Demographics**						
Age						
Mean (S.D.)	30.3 (1.8)	0.734	56 (6.2)	0.254	38.9 (12.9)	0.609
Marital status						
Single/divorced/widow	50 (70.4)	0.348	8 (72.7)	0.931	58 (70.7)	0.550
Married/cohabited	36 (78.3)		35 (71.4)		71 (74.7)	
Highest Education attainment						
Primary or below	0 (0.0)	N/A	6 (75.0)	0.913	6 (75.0)	0.900
Secondary	0 (0.0)		27 (69.2)		27 (69.2)	
Tertiary or above	86 (73.5)		10 (76.9)		96 (73.8)	
Religious Belief						
Yes	30 (88.2)	0.022 ^#^	15 (65.2)	0.382	45 (78.9)	0.211
No	56 (67.5)		28 (75.7)		84 (70.0)	
Employment status						
Full-time/part-time	86 (74.1)	0.265	19 (86.4)	0.135	105 (76.1)	0.155
Unemployed	0 (0.0)		3 (75.0)		3 (75.0)	
Retired/Housewife	0 (0.0)		21 (61.8)		21 (60.0)	
**Lifestyle behaviour**						
Age at first sexual intercourse						
≤17	8 (88.9)	0.680	4 (100.0)	0.566	12 (92.3)	0.300
≥18	66 (78.6)		39 (70.9)		105 (75.5)	
Number of lifetime sexual partner						
≥2	31 (79.5)	0.987	16 (80.0)	0.539	47 (79.7)	0.251
≤1	51 (72.9)		27 (69.2)		78 (71.6)	
Frequent sexual activity ^&^						
Active	54 (81.8)	0.400	12 (66.7)	0.446	66 (78.6)	0.646
Occasional	20 (74.1)		29 (76.3)		49 (75.4)	
Condom use ^&^						
Never/Sometimes	31 (75.6)	0.516	17 (65.4)	0.534	48 (71.6)	0.141
Always/Often	43 (82.7)		9 (81.8)		52 (82.5)	
Post-coital bleeding ^&^						
Yes	12 (100.0)	0.117	2 (50.0)	0.305	14 (87.5)	0.522
No	62 (76.5)		24 (75.0)		86 (76.1)	
Urethritis ^&^						
Yes	10 (90.9)	0.284	7 (77.8)	0.720	17 (85.0)	0.286
No	76 (71.7)		36 (72.0)		112 (71.8)	
STD						
Yes	0 (0.0)	N/A	3 (100.0)	0.551	3 (100.0)	0.564
No	86 (73.5)		40 (70.2)		126 (72.4)	
Screening patterns (Pap)						
Never/Under-screened	48 (72.7)	0.916	22 (78.6)	0.234	71 (74.7)	0.517
Regularly screened	37 (74.0)		20 (64.5)		57 (70.4)	

* Chi-square/Fisher exact tests were used for categorical variables; # *p*-Value < 0.05; ^&^ Data referred to the past 2 years among community respondents, whereas in the past 6 months among vaccinated females. Missing values are not presented. % Column percentage.

**Table 3:** Comparison of perception on handling HPV self-sampling between the two age groups.

Perception of Handling Self-Sampling	Aged 25–35 N = 86 (%) ^	Aged ≥45 N = 39 (%) ^	*p*-Value
Ease of use			
Easy to perform	57 (66.3)	32 (82.1)	0.071
Easy to handle	67 (77.9)	33 (84.6)	0.385
Handling procedure			
To collect self-samples correctly	51 (59.3)	26 (66.7)	0.433
Confidence in handling procedure	69 (80.2)	32 (82.1)	0.811
Beneficial to health	81 (94.2)	37 (94.9)	0.877
Convenience	74 (86.0)	37 (94.9)	0.058
Trusting the test results	62 (72.1)	29 (74.4)	0.792

^ percentage of women who scored 4 or above on the Likert scale from 1—strongly disagree to 5—strongly agree.
